# Longitudinal Associations of Substance Use Risk Profiles with the Use of Alternative Tobacco Products and Conventional Smoking among Adolescents

**DOI:** 10.3390/ijerph182413248

**Published:** 2021-12-16

**Authors:** Marieke Hiemstra, Andrea Rozema, Maria Jansen, Hans van Oers, Jolanda Mathijssen

**Affiliations:** 1 Academic Collaborative Centre for Public Health Brabant, Department Tranzo, Tilburg University, 5000 LE Tilburg, The Netherlands; a.d.rozema@tilburguniversity.edu (A.R.); j.a.m.vanoers@tilburguniversity.edu (H.v.O.); j.j.p.mathijssen@tilburguniversity.edu (J.M.); 2 National Institute for Public Health and the Environment (RIVM), 3720 BA Bilthoven, The Netherlands; 3 Academic Collaborative Centre for Public Health Limburg, Public Health Service South Limburg (GGD ZL), 6400 AA Geleen, The Netherlands; maria.jansen@ggdzl.nl; 4 Department of Health Services Research, Care and Public Health Research Institute (CAPHRI), Maastricht University, 6211 LK Maastricht, The Netherlands

**Keywords:** tobacco use, conventional smoking, e-cigarette, shisha-pen, water pipe, personality traits, adolescents

## Abstract

Although personality is associated with the onset of substance use (i.e., conventional smoking, alcohol use, and cannabis use) during adolescence, it is unclear whether personality traits are also associated with the onset of use of alternative tobacco products (ATPs), i.e., electronic cigarettes, shisha-pens, and water pipes. This study examines whether personality traits are associated with the onset of use of both conventional cigarettes and ATPs. Longitudinal data (baseline and 18-month follow-up) were used. The sample consisted of 1114 non-user adolescents (mean age = 13.36, SD = 0.93, 56% female) at baseline. To measure personality traits, the Substance Use Risk Profile Scale was used with four subscales: anxiety sensitivity, hopelessness, sensation seeking, and impulsivity. Structural equation models were conducted using Mplus 7.3. Results showed that both hopelessness and sensation seeking were associated with the onset of use of e-cigarettes and conventional cigarettes. Further, sensation seeking and impulsivity were associated with the onset of use of shisha-pens and water pipes. In conclusion, to prevent adolescents from using ATPs and/or conventional cigarettes, it is important to take their personality traits into account. More research on other (shared) risk factors and on more advanced stages of ATP use is needed before effective prevention strategies can be developed.

## 1. Introduction

Whereas in the last century the use of ‘conventional’ cigarettes decreased in most Western countries, alternative tobacco products (ATPs) have increased in popularity in recent years (e.g., [1,2]). ATPs include electronic cigarettes (e-cigarettes with nicotine), shisha-pens (i.e., e-cigarettes without nicotine, which mimic the taste of a water pipe [3,4]), and water pipes (also known as ‘shishas’ or ‘hookahs’). Among youth in the U.S., e-cigarettes (including shisha-pens) are the most commonly used tobacco products (20.8%), with their use currently exceeding the use of conventional cigarettes (8.1%) [1]. The same trend is seen in the Netherlands. In 2019, 24.8% of adolescents aged 12–16 years had ever smoked e-cigarettes (including shisha-pens, e-hookahs, e-smokers, and flavor vape), while only 16.7% had ever smoked conventional cigarettes [4]. Current water pipe use among adolescents was 4.1% in the U.S. [5]. In the Netherlands, 17% of adolescents said that they had used a water pipe at least once [4]. Although e-cigarettes were originally developed to aid conventional smoking cessation in adults, recent studies (e.g., [6,7,8,9]) have shown that e-cigarettes can act as a gateway to conventional smoking among adolescents. This was also found for shisha-pen use [10]. For adolescents, e-cigarettes are an attractive alternative because e-cigarettes are less harmful and they are available in many different flavors (i.e., shisha-pen) [11]. With the emerging use of ATPs, it is important to prevent adolescents from starting to use ATPs besides conventional smoking. To develop effective preventive interventions, greater understanding of the mechanisms underlying alternative product use among adolescents is required. An important factor in the onset of conventional smoking is personality [12,13,14,15]. Commonly studied personality traits in substance use are (i) anxiety sensitivity (i.e., fear of symptoms of physical arousal [16]); (ii) hopelessness (i.e., characteristics of depressive feelings or negative thinking [17]); (iii) sensation seeking (i.e., craving for intense and novel experiences and risk taking [18]); and (iv) impulsivity (i.e., difficulties in controlling behavioral responses [19]) (e.g., [20]).

Many studies have focused on the relationship between personality traits and conventional cigarette use. Recently, Ali et al. [21] presented an overview of studies focusing on personality traits and substance use in different samples of adolescents. In that review, results from longitudinal adolescent studies focusing on conventional smoking showed that anxiety sensitivity was not associated with conventional smoking (e.g., [22,23,24,25]). For hopelessness, most adolescent studies showed positive relationships: e.g., hopelessness was associated with more conventional smoking (e.g., [22,23]). In addition, a higher level of sensation seeking was associated with more tobacco use ([23,24,25,26]). Finally, impulsivity was also found to be positively associated with tobacco use in longitudinal adolescent studies (e.g., [22,23,25,27]).

Although there is an extensive body of research on conventional smoking, research on the role of personality traits in adolescent ATP use is limited. Of the few studies that have investigated the link between personality traits and ATP use among adolescents, most focus on sensation seeking and e-cigarettes. For example, sensation seeking was associated with e-cigarette use in bivariate analysis, whereas in the multivariate analysis the association was no longer significant in a student sample [28]. In another cross-sectional young-adult study, sensation seeking was associated with both ever and current e-cigarette use [29]. Longitudinal studies have also shown that higher sensation seeking is associated with e-cigarette use in adolescent samples, e.g., [30]. Moreover, various studies have shown that impulsivity is associated with e-cigarette use in adolescents [31] and young adults [29,32,33].

To our knowledge, no studies have focused on anxiety sensitivity or hopelessness and e-cigarette use during adolescence. Furthermore, in the above-mentioned studies, no distinction was made between e-cigarettes and shisha-pens. Researching both separately will provide valuable in-depth information. For water pipe use, one study showed that impulsivity is associated with hookah tobacco use [34]. Overall, little is known about the effects of personality traits on the onset of ATP use.

**Figure 1:**
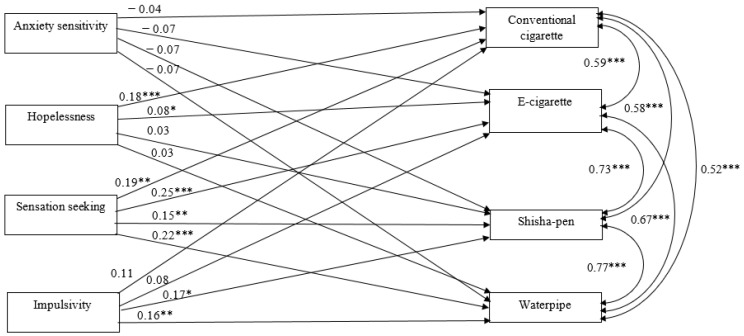
Standardized estimates (β) of relationship between SURPS at baseline and substance use onset at 18-months follow-up (*n* = 1032). Note: in this model, the following covariates were controlled for: sex, ethnicity, educational level, grade, and outdoor school smoking policy. * *p* < 0.05; ** *p* < 0.01; *** *p* < 0.001.

The aim of the present longitudinal study was to examine the relation between personality traits and adolescents’ use of ATPs and conventional smoking. Based on the literature, we hypothesized that a higher score on hopelessness, sensation seeking, and impulsivity would be associated with higher onset of use of alternative smoking products and conventional smoking 18 months later. Based on the literature for anxiety sensitivity, no such association was expected. Since ATP use and conventional tobacco use are highly associated (e.g., [35]), these outcomes were tested simultaneously in one model (Figure 1).

## 2. Materials and Methods

### 2.1. Procedure and Participants

The data for this study were collected as part of a broader effectiveness study on the effect of outdoor school ground smoking bans on conventional and alternative smoking behavior of adolescents. A complete description of the study is available elsewhere [36]. Briefly, 19 Dutch schools participated and informed consent was obtained from all participants. As students were of minority age (<18 years), their parents were fully informed beforehand about the study. Some parents (*n* = 30) refused to give parental consent and these students did not participate in the study. The study was approved by the Psychological Ethics Committee of Tilburg University (EC-2014.19). 

Adolescent questionnaire data were collected during school hours. Baseline data were collected between March 2014 and May 2015; the 18-month follow-up data were collected between October 2015 and January 2017. At both waves, adolescent questionnaires were administered at school online, or on paper under the supervision of a teacher (paper questionnaire: 16% at baseline; 43% at 18-month follow-up). To overcome the possible interference of intervention effects, all the analyses controlled for the outdoor smoking policy of the individual schools (36.7% no outdoor smoking ban; 63.3% outdoor smoking ban). For more information about the implementation of the outdoor smoking policy, see [36].

#### Attrition Analyses

Overall, of the 5742 adolescents who participated at baseline, 1618 (28.2%) also participated at the 18-month follow-up. The relatively high dropout rate might be (partly) explained by the fact that three schools with the no outdoor smoking ban condition ceased participation during the study periods (*n* = 1141). Attrition analysis comparing adolescents who participated at baseline and at the 18-month follow-up (*n* = 1618) compared to drop out (*n* = 4124) showed that girls were less likely to drop out than boys (OR = 0.83, 95%CI = 0.72–0.95, *p* < 0.01) (see also Table 1). Further, migrant adolescents were more likely to drop out compared with native adolescents (OR = 1.47, 95%CI = 1.237–1.77, *p* ≤ 0.001); adolescents in higher grades at schools were more likely to drop out compared to the lower grades (OR = 3.51, 95%CI = 3.06–4.04, *p* ≤ 0.001), and higher educated adolescents were more likely to drop out compared to lower educated adolescents (OR = 1.18, 95%CI = 1.10–1.27, *p* ≤ 0.001). Older adolescents were less likely to drop out than younger adolescents (OR = 0.70, 95%CI = 0.63–0.77, *p* ≤ 0.001). Moreover, drop outs more often used a water pipe (OR = 1.38, 95%CI = 1.10–1.72, *p* < 0.01). No differences were found for use of e-cigarettes, shisha-pens, or conventional smoking. For the personality traits, differences were only found for sensation seeking: adolescents who scored high on sensation seeking were less likely to drop out (OR = 0.89, 95%CI = 0.79–0.99, *p* < 0.05). No differences in dropout rate were found for the condition (outdoor school smoking policy versus no outdoor school smoking policy).

For the present study, only adolescents were included who completed the baseline and 18-month follow-up measurements and were non users at baseline, to test effects on the onset of use (*n* = 1114; 19.4% of the original sample).

### 2.2. Sample Characteristics

Most of the adolescents were of Dutch origin (86.4%). The adolescents’ mean age at baseline was 13.36 (*SD* = 0.93; range 11–16) years and 43.9% were boys (see also Table 1). Regarding educational level at baseline, 43% of youths followed lower education (i.e., schools specialized in students with learning difficulties and pre-vocational secondary education), 26.7% followed average education (i.e., lower general secondary education), 13.4% followed middle education (i.e., higher general secondary education), and 17.0% followed higher education (i.e., pre-university education). At baseline, the participating adolescents were in grades 7–9 (7th grade 49.8%; 8th grade 40.4%; 9th grade 9.8%).

### 2.3. Measures

Personality profiles were measured at baseline with the Dutch translation of the Substance Use Risk Profile Scale (SURPS) [15,20]. The SURPS measures four personality traits: anxiety sensitivity, hopelessness, sensation seeking, and impulsivity. Each trait was measured with 5–7 items (in total 23 items) that could be answered on a 4-point scale ranging from 1 = strongly agree to 4 = strongly disagree. Example items were: ‘It’s frightening to feel dizzy or faint’ for anxiety sensitivity, ‘I’m happy’ for hopelessness, ‘I like doing things that frighten me a little’ for sensation seeking, and ‘I usually act without stopping to think’ for impulsivity. Cronbach’s alphas were 0.76 for anxiety sensitivity, 0.85 for hopelessness, 0.72 for sensation seeking, and 0.74 for impulsivity. The reliability and validity of the instrument are adequate (e.g., [20,23]).

Conventional cigarettes: Adolescents’ smoking status for conventional cigarettes was assessed at each wave. Adolescents were asked to report, on a 5-point scale, which stage of conventional smoking applied to them. Response categories ranged from 1 ‘never smoked’, 2 ‘smoked only once or twice’, 3 ‘smoke occasionally, but not every day’, 4 ‘smoked in the past’, and 5 ‘smoke every day’. Adolescents who ‘never smoked a cigarette’ were coded as ‘non-users’. Those who ‘smoked a cigarette once or twice’, ‘smoke occasionally, but not every day’, ‘smoked in the past’, and ‘smoke every day’ were coded as ‘users’. For conventional smoking onset, only adolescents who had never smoked at baseline were included in the analyses (*n* = 1105).

Alternative tobacco products: Adolescents were asked the following question ‘How old were you when you used this substance/device for the first time?’, with answer categories ‘I never used this substance/device’, ’11 years or younger’, ’12 years’, ’13 years’, ’14 years’, ’15 years’, ’16 years’, ’17 years’, ’18 years’, ’19 years’, and ’20 years or older’ for (i) e-cigarettes, (ii) shisha-pens (or electronic waterpipe), and (iii) water pipes. Adolescents who responded ‘I never used this substance/device’ were classified as 0 ‘non users’ and students who responded with an age at which they used the substance for the first time were classified as 1 ’users’. These ATPs were measured because we expected that these products were most popular in the Netherlands. Only adolescents who were non users at baseline were included in the analyses (e-cigarette *n* = 1074; shisha-pen *n* = 1073; water pipe *n* = 1072) to look for the onset of ATP use. Sample sizes for the different ATPs differ because of missing response(s) on some substances.

### 2.4. Statistical Analysis

Descriptive statistics and Pearson’s correlations were calculated using SPSS 22.0 (SPSS Inc., Chicago, IL, USA). To examine whether the baseline substance use risk profiles (i.e., social anxiety, hopeless, sensation seeking, and impulsivity) were related to the onset of conventional cigarette use, e-cigarette use, shisha-pen use, and water pipe use 18 months later, structural equation models (SEM) were tested with Mplus 7.3 (Mplus, Los Angeles, CA, USA) [37]. Sex, ethnicity, grade, educational level at baseline, and outdoor school smoking policy were specified as covariates in the model. 

The model included a mixture of categorical and continuous variables (Figure 1). Conventional smoking and ATP variables were categorical (binary), whereas the others were continuous. Mplus allows the use of both continuous and categorical variables as independent and dependent variables. As a special case of SEM, path analysis with a categorical dependent variable was used [37]. In the model, the covariates and correlations between substance use were controlled for. With Mplus, the correlation matrix of these variables and the parameters in the model were estimated according to the weighted least square method with adjusted mean and variance chi-square statistics (WLSMV estimator). The fit of both models was assessed using the following fit indices: χ2, comparative it index (CFI) (with a cut-off value of 0.95) and root mean square error of approximation (RMSEA) (with a cut-off value of 0.06) [37,38].

The data were nested within schools (*n* = 16); we corrected for this via the CLUSTER command in combination with the TYPE = COMPLEX procedure in Mplus [37]. This method corrects for dependency, which leads to unbiased standard errors of the estimated parameters.

## 3. Results

### 3.1. Descriptives and Pearson’s Correlations

Of the 1114 non-using adolescents at baseline, 30.1% had started conventional smoking or ATP use by the 18-month follow-up. With regard to the different products, 184 (16.7%) had started using conventional cigarettes, 83 (7.7%) e-cigarettes, 192 (17.9%) shisha-pens, and 134 (12.5%) water pipes. The overlap of use between conventional smoking and ATP use was as follows: 6.7% were conventional smokers, 13.6% only used ATPs, and 9.7% used both. Overall, Table 2 shows participant characteristics of the users and non-users (conventional smoking and/or ATP use) at 18-month follow-up. Table 3 shows the Pearson’s correlations between the model variables. More specifically, Pearson’s correlations showed that, at 18-month follow-up: (i) anxiety sensitivity was not associated with the onset of conventional cigarette or ATP use, (ii) hopelessness was only associated with conventional cigarette use, and (iii) sensation seeking and impulsivity were associated with conventional cigarette, e-cigarette, shisha-pen, and water pipe use.

### 3.2. Personality Traits and Substance Use Onset

The model depicted in Figure 1 shows a good fit to the data [χ2 (df = 42, *n* = 1032) = 67.46, CFI = 1.00, RMSEA = 0.00]. No significant association was found between anxiety sensitivity and onset of conventional smoking or ATP use. Hopelessness was positively associated with onset of conventional cigarette use (β = 0.18 (0.04), *p* < 0.001) and e-cigarette use (β = 0.08 (0.04), *p* < 0.05); adolescents with higher levels of hopelessness at baseline were more likely to start using conventional cigarettes and e-cigarettes 18 months later. Sensation seeking was positively associated with conventional smoking and the use of all ATPs (conventional cigarettes: β = 0.19 (0.07), *p* ≤ 0.01; e-cigarettes: β = 0.25 (0.06), *p* ≤ 0.001; shisha-pens: β = 0.15 (0.05), *p* ≤ 0.01, water pipes: β = 0.22 (0.05), *p* ≤ 0.001). Thus, adolescents with a high level of sensation seeking at baseline were more likely to start using conventional cigarettes, e-cigarettes, shisha-pens, and water pipes. Impulsivity was positively associated with shisha-pen use (β = 0.17 (0.08), *p* ≤ 0.05) and water pipe use (β = 0.16 (0.05), *p* ≤ 0.01); the more impulsivity at baseline, the more likely the use of shisha-pens and water pipes 18 months later.

For the covariates in the model, a significant relationship was found between educational level and conventional smoking (β = −0.19 (0.06), *p* ≤ 0.001) and e-cigarette use (β = −0.23 (0.07), *p* ≤ 0.01); the higher the level of education, the less likely the onset of conventional smoking and the use of e-cigarettes. Outdoor school smoking policy was associated with water pipe use (β = 0.19 (0.09), *p* ≤ 0.05); adolescents in the outdoor smoking ban condition were more likely to start using water pipes than those in the no outdoor smoking ban condition. Sex was associated with conventional smoking (β = 0.10 (0.04), *p* ≤ 0.05); girls more often started smoking than boys. For the other covariates, no significant relationships were found with conventional smoking or the use of ATPs. For the smoking outcomes, we found that they were strongly related with each other (β = 0.52–0.77, *p* ≤ 0.001; Figure 1). The model showed small to medium effect sizes (Cohen, 1992). The explained variance was 17.5% for conventional smoking onset, 12.7% for e-cigarette use, 8% for shisha-pen use, and 16.1% for water pipe use.

## 4. Discussion

The present study tested whether personality traits (i.e., anxiety sensitivity, hopelessness, sensation seeking, and impulsivity) are associated with the onset of the use of ATPs and conventional cigarettes among adolescents. In summary, the results show that higher levels of hopelessness and higher levels of sensation seeking are associated with the onset of the use of conventional cigarettes and e-cigarettes. Higher levels of sensation seeking and higher levels of impulsivity increase the likelihood of the onset of use of shisha-pens and water pipes.

The results regarding conventional smoking and the use of e-cigarettes are in line with previous studies in which sensation seeking was an important predictor of conventional smoking onset (e.g., [23]) and e-cigarette use (e.g., [28]). Sensation seekers tend to have a ‘novelty seeking’ nature, which can lead to experimentation with new tobacco products.

The results regarding hopelessness and conventional smoking are also in line with the results of previous studies [22,23,24]. However, we also found a relationship between hopelessness and the onset of e-cigarette use. A possible explanation for this relationship is that starting to use e-cigarettes might be a way of dealing with depressive feelings or negative thinking. For impulsivity, only bivariate Pearson’s correlations for both conventional smoking and e-cigarette smoking were found, indicating that (besides hopelessness and sensation seeking) the effect of impulsivity was not strong enough. A possible explanation for this might be found in neurobiological theories (e.g., [39]) that suggest that sensation seeking is related to increased dopamine levels in the reward system [40] and impulsivity is related to inhibition control. Thus, impulsivity might be more important in the maintenance of substance use, and sensation seeking in the onset of use. Future studies could focus on these items and on more advanced stages of e-cigarette use.

Important personality traits for both shisha-pen and water pipe use were impulsivity and sensation seeking. An explanation for the effect found for sensation seeking is similar to that for conventional smoking and e-cigarette use: sensation seekers generally like to try new things, such as shisha-pens or water pipes. An explanation of why impulsivity is important in the onset of water pipe use might be that using a water pipe is a social activity that can be shared with friends [41]. Moreover, adolescents under peer pressure show more impulsive behavior (e.g., [42]).

As expected, anxiety sensitivity showed no significant relationship with the use of any of the tobacco products. This might be because anxiety sensitive individuals are generally more worried/bothered about trying new things.

Previous studies made no distinction between e-cigarettes with and without nicotine (shisha-pens); however, the present study shows that the personality traits associated with shisha-pen use are more similar to those of water pipe use than to the use of e-cigarettes with nicotine. Personality traits associated with the use of e-cigarettes are similar to those associated with conventional cigarette use. A reason for this could be that e-cigarettes were developed as a substitute for conventional cigarettes and attracted a similar type of person (e.g., more sensitive to addiction); this knowledge might be important when developing prevention strategies. More research is needed on the overlap of risk factors associated with the onset of conventional smoking and the different ATPs, such as genetic risk factors and cognition, to better understand why adolescents start using any type of smoking products [43]. To our knowledge, this is the first study to evaluate longitudinal relationships between personality traits and ATP use in a large sample of adolescents. The results suggest that personality traits should be taken into consideration in intervention programs focusing on substance use, such as the effective Preventure program. In this program, adolescents are selected based on personality scales (often using SURPS) and receive a targeted intervention program focusing on their personality traits with cognitive behavior therapy and motivational interviewing [44]. 

However, the study has some limitations. First, adolescents self-reported their conventional smoking and ATP use; this might introduce under or over-reporting due to recall bias and/or social desirability. However, self-reported data on conventional smoking are generally reliable when confidentiality is assured (e.g., [45]). Second, there was substantial dropout. Therefore, some caution is warranted when interpreting and generalizing the findings. However, the prevalence rates of tobacco product use in the included sample of the present study were similar to those of a representative Dutch study among adolescents [46]. Nevertheless, the present study focused on the onset of ATP use. If comparable with conventional smoking, ATP use will probably develop through various stages, from trying out to daily use [47], in which different risk factors become important at more advanced stages of ATP use. Therefore, future studies should focus on different stages of ATP use, and associated risk factors (e.g., personality traits, friends’ e-cigarette use, and cognition) should be tested. 

## 5. Conclusions

In conclusion, the present findings suggest that, during early adolescence, different personality traits are associated with the onset of ATP use and conventional smoking. This knowledge might be useful for developing effective prevention strategies for ATP use. However, more research is needed that focuses on more advanced stages of ATP use and shared risk factors.

## Figures and Tables

**Table 1:** Participants’ characteristics. Values are percentages unless stated otherwise.

	Participants at Baseline (*n* = 5742)	Participants Participating at Baseline and 18 Months Follow-Up (*n* = 1618, 28.2%)	Non-Users at Baseline Participating at 18 Months Follow-Up (*n* = 1114, 19.4%)	Attrition Alyses **
Sex				OR = 0.83, 95%CI = 0.72–0.95, *p* < 0.01
Boys	51.8	49.1	43.9	
Girls	48.2	50.9	56.1	
Mean age of participants (Mean (SD)) (range)	13.74 (1.10) (11–18)	13.45 (0.94) (11–16)	13.36 (0.93) (11–16)	OR = 0.70, 95%CI = 0.63–0.77, *p* ≤ 0.001
Origin				OR = 1.47, 95%CI = 1.237–1.77, *p* ≤ 0.001
Dutch	82.1	85.3	86.4	
Other	17.9	14.7	13.6	
Education *				OR = 1.18, 95%CI = 1.10–1.27, *p* ≤ 0.001
Lower	32.0	45.5	43.0	
Average	33.1	27.3	26.7	
Middle	18.7	12.5	13.4	
Higher	15.7	14.7	17.0	
Grade				OR = 3.51, 95%CI = 3.06–4.04, *p* ≤ 0.001
7th	30.0	45.2	49.8	
8th	32.6	42.9	40.4	
9th	35.8	11.7	9.8	
Outdoor school smoking policy				n.s
No outdoor smoking policy	35.6	36.7	38.2	
Outdoor smoking policy	64.4	63.3	61.8	
Personality Profiles				
Anxiety sensitivity (Mean (SD))	1.99 (0.72)	1.98 (0.70)	1.98 (0.68)	n.s.
Hopelessness (Mean (SD))	1.52 (0.58)	1.51 (0.56)	1.45 (0.53)	n.s.
Sensation seeking (Mean (SD))	2.55 (0.72)	2.52 (0.70)	2.42 (0.70)	OR = 0.89, 95%CI = 0.79–0.99, *p* < 0.05
Impulsivity (Mean (SD))	2.05 (0.68)	2.03 (0.66)	1.93 (0.64)	n.s.
Baseline substance use				
Conventional cigarette	21.6	15.9	0.0	n.s.
E-cigarette	13.4	10.6	0.0	n.s.
Shisha-pen	28.6	22.4	0.0	n.s.
Water pipe	21.4	14.5	0.0	OR = 1.38, 95%CI = 1.10–1.72, *p* < 0.01
Follow-up substance use				
Conventional cigarette	28.4	28.4	16.7	-
E-cigarette	15.5	15.5	7.7	-
Shisha-pen	28.9	28.9	17.9	-
Water pipe	24.9	24.9	12.5	-

* Lower education are schools specialized in students with learning difficulties and pre-vocational secondary education, average education is lower general secondary education, middle education is higher general secondary education, and higher education is pre-university education; ** attrition analyses were between participants who participated at baseline and between participants who participated at baseline and 18 months; OR = Odds ratio; 95%CI = 95% Confidence Interval.

**Table 2:** Participant characteristics of non-users versus users (conventional smoking and/or ATP use) at 18 months follow up (*n* =1114).

Variables ^$^	Non-Users at 18 Months (69.9%)	Users ^#^ at 18 Months Follow-Up (30.1%)
Sex		
Girls	56.9	53.6
Boys	43.1	46.4
Mean age of participants (Mean (SD)) (range)	13.34 (0.93) 11–16	13.41 (0.93)
Origin		
Dutch	86.6	89.4
Other	13.4	10.6
Education		
Lower	40.8	48.3
Average	25.1	28.2 *
Middle	14.3	11.6 *
Higher	19.8	11.9
Grade		
7th	50.5	47.8
8th	40.4	40.7
9th	9.0	11.5
Outdoor school smoking policy		
No outdoor smoking policy	42.3	30.4 **
Outdoor smoking policy	57.7	69.6
Personality Profiles at baseline		
Anxiety sensitivity (Mean (SD))	1.97 (0.67)	2.02 (0.71)
Hopelessness (Mean (SD))	1.42 (0.51)	1.52 (0.55) *
Sensation seeking (Mean (SD))	2.34 (0.69)	2.61 (0.68) ***
Impulsivity (Mean (SD))	1.84 (0.60)	2.13 (0.68) ***

^#^ 15.1% used 1 product, 8.9% used 2 products, 3.2% used 3 products and 3.0% used all 4 products; ^$^ For categorical variables, the first category was reference class; significant differences between non-users and users were tested with logistic regression analyses; * *p* < 0.05; ** *p* < 0.01; *** *p* < 0.001.

**Table 3:** Bivariate correlations of conventional cigarettes, ATPs, and the four personality traits.

	1	2	3	4	5	6	7	8
1. Conventional cigarette (follow-up)	-							
2. E-cigarette (follow-up)	0.33 ***	-						
3. Shisha-pen (follow-up)	0.34 ***	0.43 ***	-					
4. Water pipe (follow-up)	0.31 **	0.38 ***	0.49 ***	-				
5. Anxiety sensitivity (baseline)	0.05	0.03	0.01	0.02	-			
6. Hopelessness (baseline)	0.16 ***	0.04	0.01	0.03	0.11 ***	-		
7. Sensation seeking (baseline)	0.13 ***	0.14 ***	0.15 ***	0.19 ***	0.16 ***	−0.15 ***	-	
8. Impulsivity (baseline)	0.16 ***	0.12 ***	0.14 ***	0.16 ***	0.39 ***	0.13 ***	0.47 ***	-

*** *p* < 0.001, ** *p* < 0.01.

## Data Availability

Data available on request due to restrictions eg privacy or ethical. The data presented in this study are available on request form the corresponding author. The data are not publicly available due to fact that there was not asked for permission in the informed consent.
